# Juvenile King Scallop, *Pecten maximus*, Is Potentially Tolerant to Low Levels of Ocean Acidification When Food Is Unrestricted

**DOI:** 10.1371/journal.pone.0074118

**Published:** 2013-09-04

**Authors:** Matthew Burton Sanders, Tim P. Bean, Thomas H. Hutchinson, Will J. F. Le Quesne

**Affiliations:** 1 Centre for Environment, Fisheries & Aquaculture Science, Weymouth, Dorset, United Kingdom; 2 Centre for Environment, Fisheries & Aquaculture Science, Lowestoft Laboratory, Lowestoft, Suffolk, United Kingdom; University of Gothenburg, Sweden

## Abstract

The decline in ocean water pH and changes in carbonate saturation states through anthropogenically mediated increases in atmospheric CO_2_ levels may pose a hazard to marine organisms. This may be particularly acute for those species reliant on calcareous structures like shells and exoskeletons. This is of particular concern in the case of valuable commercially exploited species such as the king scallop, *Pecten maximus*. In this study we investigated the effects on oxygen consumption, clearance rates and cellular turnover in juvenile *P* *. maximus* following 3 months laboratory exposure to four *p*CO_2_ treatments (290, 380, 750 and 1140 µatm). None of the exposure levels were found to have significant effect on the clearance rates, respiration rates, condition index or cellular turnover (RNA: DNA) of individuals. While it is clear that some life stages of marine bivalves appear susceptible to future levels of ocean acidification, particularly under food limiting conditions, the results from this study suggest that where food is in abundance, bivalves like juvenile *P* *. maximus* may display a tolerance to limited changes in seawater chemistry.

## Introduction

Levels of atmospheric CO_2_ could reach 730-1020 ppm by the year 2100 compared to historic levels of ~290 ppm [1], and the ocean represents a primary sink for atmospheric CO_2_. Since the 1800s the ocean has absorbed approximately 50% of anthropogenic CO_2_ [2], this reduces pH through the formation of carbonic acid (H_2_CO_3_) and its subsequent dissociation into hydrogen ions (H^+^) and bicarbonate (HCO_3_^-^); a process called ocean acidification (OA). Climate models predict that the surface ocean pH, which has already decreased by 0.1 units since the pre-industrial age [3], may decrease by a further 0.3 to 0.77 units by 2100 [4,5]. In addition to the increase in acidity, OA also reduces the saturation state of calcium carbonate (CaCO_3_), and subsequently carbonate ion availability [6].

This is of potential concern as research has shown that elevated levels of CO_2_ can affect a range of physiological processes in marine organisms including growth [7,8], feeding [9] reproduction [10,11], immune function [12] and olfactory discrimination [13]. Adverse effects are not necessarily universal, with different species demonstrating different sensitivities to OA [14–16].

Calcifying organisms were identified early as being sensitive to OA as the reduction in carbonate saturation states, arising from increasing *p*CO_2_ and decreasing pH, was predicted to impact calcification [3,6]. This is evidently true for bivalve larvae which predominantly form their shells by precipitation from the surrounding water [17]. However, larger bivalves potentially form their shell in a high *p*CO_2_ compartment which is under-saturated with respect to aragonite, even under ideal conditions [18]. Brief exposure (<1d) to seawater reduced in pH by up to 0.4 units (Δ-0.4) has been observed to adversely affects calcification rates of juvenile/adult *Mytilus* *edulis* [19], the clam *Mercenaria* *mercenaria* [20] and the oysters *Crassostrea* *gigas* [19] and *C* *. virginica* [21]. In contrast, over longer acclimation periods (>60 d) no effect on the calcification of *M* *. edulis* , *M* *. mercenaria* , *C* *. virginica* , the scallop *Argopectenirradians* [16] or the clam *Ruditapes* *decussatus* [22] has been observed in seawater reduced by as much as -0.6 pH units. Furthermore, the mussel *M* *. edulis* has been shown to successfully inhabit environments naturally under-saturated in carbonate (aragonite) and elevated in *p*CO_2_ above >2000 µatm [18]; though calcification rates appear reduced at *p*CO_2_ levels above 1400 µatm.

Bivalve molluscs have a limited capacity for acid–base regulation due to the lack of developed ion-exchange and non-bicarbonate mechanisms [23]. Consequently, their ability to regulate the acid–base status of internal tissues during contact with water elevated in *p*CO_2_ is limited. In order to correct acid–base imbalances an organism must employ energetically costly active ion exchange mechanisms [23]. Furthermore, the extracellular alterations caused by exposure to elevated *p*CO_2_ are likely to affect processes such as energy partitioning and metabolism [24]. External changes arising from elevated *p*CO_2_ have been shown to influence extracellular acid–base balance of bivalve molluscs including the mussel *M* *. edulis* [18] and the scallop *Pecten maximus* [25]. Using the mussel, *Mytilusgalloprovincialis* , Michaelidis et al. [7] demonstrated that where bivalves are unable to counter extracellular acidosis, due to elevated *p*CO_2_ in the surrounding water, a reduction in the standard metabolic rate can occur. Similar metabolic depression has been observed in *R* *. decussatus* [9] and *Chlamys nobilis* [26] exposed to elevated *p*CO_2_.

In addition to simple measures of calcification and shell growth [27], physiological changes, such as metabolic depression, are ultimately of concern as they may effect population dynamics through a reduction in growth potential or fecundity, extending time to maturity or even interfering with predator prey interaction [25]. The UK shellfish industry achieves a first sale value of £280 million per annum, with the scallop fishery alone contributing over 22% of the value [28]. A reduction in energetic performance of commercially important bivalves like scallops, oysters and mussels could have detrimental effects on marine biodiversity, marine goods and services and the sustainability of marine fisheries [29–31]. Consequently studies on the impact of OA to commercially important calcifying organisms should consider potential effect and physiological trade-offs on whole organism fitness through measures such as feeding, energy assimilation and growth potential.

Energy assimilation and growth potential is commonly assessed in bivalves by scope for growth assessment which is dominated by the ingestion rate, derived from clearance rates, and metabolic O_2_ demand [32–35]. In a recent review, Gazeau et al. [36] highlighted that the standard metabolic rate alone could be a major factor in determining an organism’s ability to tolerate the effects of OA. Additional assessment of RNA: DNA ratios can also provide a measure of nutritional condition and growth potential within the animal [37].

Marine bivalves inhabiting the intertidal region experience changes in body fluid *p*CO_2_ levels during periods of emersion (due to respiration) [38] and may be pre-adapted to utilise metabolic depression as a response to elevated *p*CO_2_ [36,39]. Such pre-adaption to fluctuations could provide a mechanism for coping with changes in *p*CO_2_ levels due to ocean acidification. Significant compensatory mechanisms have recently been identified in the gene expression profiles of *M* *. edulis* exposed to 385-4000 µatm CO_2_ [40]. Interpreting such responses is difficult without first understanding the physiological effect of acidification on the whole organism. In contrast to intertidal organisms, sub-tidal marine bivalves, like the scallop *Pecten maximus*, do not naturally experience internal *p*CO_2_ fluctuations due to periods of emersion. Consequently, such species may not readily employ metabolic depression as a response mechanism and could be more sensitive to elevated *p*CO_2_ from OA.

This study examined the impact of OA on the commercially valuable bivalve mollusc, *P* *. maximus* , with mid-term exposures (11 weeks) across a range of CO_2_ exposures before attempting investigation and comprehension of sub-cellular responses. The aim was to assess the impact of OA on the growth potential and possible energetic trade-offs in scallops, beyond measures of calcification, by assessment of the feeding rate, respiration rate, condition index and RNA: DNA ratios as tools for assessing impacts of OA at the whole organism level. 

## Materials and Methods

### Scallop source and husbandry

Juvenile *Pecten maximus* were supplied by Northwest Shellfish (Ireland) in February 2011. In the morning scallops were hand graded at site to ~50mm shell height, wrapped in macro algae and immediately transported to Cefas on ice within 12h. Epibionts were removed from the shells and the scallops initially held in 300 L tanks supplied with ambient seawater at around 390 parts per million CO_2_ by volume (ppmv) at 10 °C. To mitigate potential temperature dependent quiescence [41] and to allow comparisons of clearance rates with previous studies [42], the temperature was raised to 15 °C at 0. 5 °C d^-1^ over 10 days before the study. Scallops were fed a 2% maintenance ration (dry weight algae/ wet weight scallop flesh) Shellfish Diet 1800 composed of 40% *Isochrysis* spp, 25% *Tetraselmis* spp, 15% *Pavlova* spp, 20% *Thalassiosira* *weissflogii* according to manufacturer’s instructions (Reed Mariculture, USA). After a further 10 d at the acclimated temperature, 210 juvenile scallops were individually tagged and the shell height recorded to 0.1 mm with callipers (Mitutoyo Corp., Japan). The scallops were returned to the stock tanks for a further 2 days prior to allocation to experimental tanks. There were no mortalities during the acclimation holding period.

### Experimental facilities

The experiment was conducted at the ocean acidification experimental facility at Cefas in Weymouth, United Kingdom (UK) using methodology similar to Roberts et al. [43]. In brief, ambient air was compressed, filtered and dried (MMP140, Millenium Medical Products UK) and the CO_2_ content measured using a calibrated GMZ 750 IRA analyser (UMSITEC, Germany). CO_2_ (certified 99.5% food grade, BOC) was added via paired Red-y smart meter and controller (Vogtlin Instruments, Germany) to achieve the desired gas mixtures of 290, 390, 750 and 1140 µatm CO_2_ [44]. Natural seawater (salinity approximately 35 psu) from the English Channel was supplied by a coastal inlet pipe to the laboratory and passed through a 0.45 µm filter and UV sterilisation prior to modification. The seawater was pH modified by passing sterilised seawater vertically downward through 4 interlinked columns (0.2 m Ø x 2.2 m) against a countercurrent gas flow added through the base of each column via a ceramic fine bubble diffuser (FBS-775; Diffused Gas Technologies, USA) equipped with an AF30 head (0.19 m diameter, 22 µm pore size). Gas flow to each diffuser was individually controlled by a Q-Flow 140 flow meter (Vogtlin Instruments, Switzerland). An additional bleed valve permitted independent measurement of gas mixtures using a CO_2_/H_2_O gas analyser (LI-840, LI-COR, Nebraska, USA) calibrated against certified CO_2_ gas mixtures (0 and 2000 ppmv, BOC, UK). With an air/water contact time in excess of 3 h the flow through system was capable of producing 1.2 L of each equilibrated seawater treatment per minute.

### Exposure regime

The pre-labelled scallops were graded into 1mm size classes and randomly distributed into 8 groups of 25 scallops such that each group had a mean shell height of 46±3 mm. The 8 groups were transferred to paired polyethylene trays (30 x 50 x 8 cm), each pair receiving one of the 4 modified water supplies 290, 390, 750 or 1140 CO_2_ µatm. Water was introduced at one end of the tray at 600 ml min^-1^ and allowed to outflow at the other end. With a retention volume of 26 L, the tray exchange rate was estimated at 1.5 exchanges per hour. The trays were covered with loose fitting Perspex covers and an additional gas feed, corresponding to the treatment, was connected to an air stone in the trays to overlay water with air of the relevant *p*CO_2_. Each tank continuously received re-constituted Shellfish Diet 1800 via a peristaltic pump at 1 ml min^-1^ to achieve 5% ration (dry weight algae/ wet weight scallop flesh) over 24 hr. This equated to nominally 100 cells µl^-1^ or 4.45 mg of organic matter per litre in the exposure tanks. While phytoplankton concentrations in UK coastal waters vary greatly throughout the year, concentrations of 10-60 cells µl^-1^ are not uncommon during spring and summer months in areas inhabited by scallops [45]. Scallops in the exposure system were monitored daily and dead scallops removed.

### Monitoring pH

Temperature and pH were logged every 30 minutes by an ion electrode (8102BN; Thermo, Fisher) connected to an Orion DualStar meter (Thermo, Fisher) located in one replicate tray from each treatment. Probes were cleaned and calibrated every 2 days with potassium phosphate (pH 7.02) and borate (pH 9.98) NIST traceable buffers (Fisher Scientific). The *in situ* pH_(NBS)_ recorded by the probes was converted to pH_(seawater)_ by applying the offset derived from probe measurements of certified seawater samples (Batch 117, http://andrew.ucsd.edu/co2qc/) supplied by Andrew Dickson (Scripps Institute of Oceanography, USA). Water samples were taken weekly from each tray and passed through a 0.45 µm filter using a peristaltic pump. Salinity and temperature of each sample was recorded using a HQ40D hand held monitor (HACH, USA). Duplicate water samples were collected in a glass bottle for analysis of dissolved inorganic carbon (DIC) and total alkalinity (TA) or a plastic bottle for total phosphate (TP) and silicate (TSi) analysis. Samples were preserved with mercuric chloride and both TA and DIC analysed at the National Oceanographic Centre, Southampton, according to Dickson et al. [46]. TP and TSi were analysed according to Kirkwood [47]. The absolute pH and *p*CO_2_ was calculated on the seawater scale from TA and DIC with CO2SYS_ver. 14_ [48], employing dissociation constants fitted by Dickson and Millero [49] and KSO_4_ from Dickson [50]. Reporting of biological effects is based on pH_(seawater)_ derived from CO2SYS.

### Sampling and Condition Index

It was necessary to stagger the sampling of the scallops at the end of the study over 2 days (day 82-84) to permit assessment of clearance and respirometry on the same animals. Twenty live scallops were sampled from each replicate tray and data was collected from individuals as summarised in Table 1. Where tissue was sampled for RNA: DNA analysis or where hemolymph was taken it was not possible to calculate condition index and these animals were all sampled on day 82. Shell data was available from all animals and included in analysis. Hemolymph was aspirated from adductor muscle by syringe prior to shucking for immune-competence assessment (data not presented). Shell height was recorded with callipers and dry weights determined after 48 h at 100 °C. Mantle, gill and adductor muscle tissue were dissected, separated into 3 replicate aliquots and snap frozen in liquid nitrogen and stored at -80 °C prior to analysis. Condition index was calculated as the ratio of dry flesh weight to dry shell weight [51].

**Table 1:** Measurements taken from 20 scallops present in each replicate tray after exposure period.

Scallop number																				
	1	2	3	4	5	6	7	8	9	10	11	12	13	14	15	16	17	18	19	20
SH	●	●	●	●	●	●	●	●	●	●	●	●	●	●	●	●	●	●	●	●
FW	●	●	●	●	●	●	●	●	●	●	-	-	-	-	-	-	-	-	-	-
SW	●	●	●	●	●	●	●	●	●	●	-	-	-	-	-	-	-	-	-	-
O_2_	●	●	●	●	●	-	-	-	-	-	-	-	-	-	-	-	-	-	-	-
CR	●	●	●	●	●	-	-	-	-	-	-	-	-	-	-	-	-	-	-	-
MT	-	-	-	-	-	-	-	-	-	-	●	●	●	●	●	●	●	●	●	●
GT	-	-	-	-	-	-	-	-	-	-	●	●	●	●	●	●	●	●	●	●
AT	-	-	-	-	-	-	-	-	-	-	-	-	-	-	-	●	●	●	●	●
H	-	-	-	-	-	-	-	-	-	-	●	●	●	●	●	-	-	-	-	-

● indicates samples taken for analysis, - no sample taken. SH = shell height, FW = dry flesh weight, SW = dry shell weight, O_2_ = respiration, CR = clearance rate, MT = mantle tissue, GT = gill tissue, AT = adductor muscle tissue, H = hemolymph.

### Clearance rates

Algal clearance rates were determined using the static method described by Widdows and Staff [34]. Prior to the study 8 scallops were allocated to individual 15 L test vessels containing 10 L of 0.2 µm filtered seawater at ambient *p*CO_2_ (~390µatm). *Isochrysis* *spp.* (Taihitian strain) was added to each vessel to achieve approximately 100 cells µl^-1^ [42]. Water samples were taken at 2 h intervals and the cell density measured using a spectromax M2 (Molecular Devices, USA; Ex440 nm; Em680 nm) calibrated against standard curves of cell counts determined using a hemocytometer. Standard curves were linear in the range 10-100 cells µl^-1^. Once scallops were shown to be filtering, indicated by declining algal counts, further algae was added to each vessel to return cell density to around 100 cells µl^-1^. Four replicate water samples were then taken from each test vessel as T=0 and at 10 minute intervals over a 90 minute period and measured against calibration data. The entire process took 2-4 h; depending on the delay in scallops initiating feeding. The clearance rate between each sampling point was calculated and the highest rate used as the maximum clearance rate [52]. The whole procedure was conducted at 16±0.5 °C in a low light room ~100 lux (LX-101; Lutron Electronic Enterprise Co, Taiwan) and the vessels aerated throughout to ensure homogenous mixing (ambient air ~390 µatm CO_2_). On days 82 and 83 the clearance rates of 5 scallops from replicate tray A or B respectively (10 per treatment) were measured using the same method as for day 0 animals, except clearance rates were assessed in pH adjusted seawater taken from the relevant treatment and aerated with relevant modified *p*CO_2_ mixtures. Individuals were then returned to the exposure system overnight prior to respirometry. Clearance rates were normalised to gram dry weight using the exponent 0.7 [53].

### Respirometry

The oxygen consumption of 8 scallops prior to the start of the study and the 5 scallops from replicate A on day 83 and replicate B on day 84 were measured in closed cell respirometers according to the static method of Widows and Staff [34]. Individuals selected for respirometry were the same individuals in which clearance rates were recorded the previous day (Table 1). In short, oxygen saturated exposure water (290, 390, 750 or 1140 µatm CO_2_) was filtered through a 0.2 µm filter and used to fill closed cell respirometry chambers containing individual scallops from the respective treatments. Pre-study scallops were measured in ambient seawater (~390 ppm). Scallops were allowed to settle for 1 h in the chamber under water of the respective treatments, prior to sealing. The lid was then attached and the O_2_ consumption in each chamber was measured over 180 minutes using a selective O_2_ electrode (model 1302; StrathKelvin Instruments, UK). Chambers were stirred and maintained at 16 ±0.4 °C throughout. Control chambers without scallops were run under identical conditions to assess microbial respiration; which constituted less than 0.5% of the O_2_ depletion observed with scallops. The scallops were then shucked and both the shell and meat dried to constant weight over 48 h at 100°C. Oxygen consumption, expressed as the resting metabolic rate (RMR) was calculated according to Schalkhausser et al. [25] and normalised to gram dry weight using the exponent 0.807, selected as representative of a range of scallop species [53,54]. Maximal oxygen depletion of up to 50% saturation was observed in some chambers where scallops displayed swimming activity and these data were excluded. Maximal oxygen consumption of resting animals was 73% O_2_ saturation but only data >80% saturation was used for calculating consumption rates.

### Biochemical indicators of growth

RNA and DNA from mantle, gill and adductor muscle tissue were extracted and measured with a method adapted from Clemmesen et al. [55] using reagents from Sigma Aldrich (Poole, UK) unless otherwise stated. Briefly, 30-50 mg of scallop tissue (mantle, adductor or gill) was added to 2 mm glass beads (Merck), 500 µL tris-SDS buffer (0.05 M Tris, 0.1 M Sodium chloride, 0.0 1M EDTA, 2% SDS, pH 8) and homogenised in a fast prep by three 45 sec bursts at 6.5 ms^-1^ over a five minute incubation period. Tris-saturated Phenol: chloroform: isoamyl alcohol (25:24:1) (500 µL) was then added to tubes before a further five minute incubation and regular mixing in fast prep. Aqueous and solvent phases were separated with ten minutes centrifugation at 17,000 g and the aqueous phase removed to a new tube and supplemented with an additional 500 µL phenol: chloroform: isoamyl alcohol (25:24:1). These were incubated at room temperature for five minutes with regular mixing then phases separated by a further five minute centrifugation at 17,000 g. Aqueous phase was removed and stored at -80^°^C until analysis. Total DNA and RNA was measured by diluting each sample 40 fold, adding 1X SYBR Gold nucleic acid stain (Invitrogen, UK) to 100 µL and measuring fluorescence with an excitation and emission of 485/535 nm in PerkinElmer 1420 Victor3 Multilabel counter. RNA was removed from this mixture by digesting RNA with 4 µg RNase A at 37^°^C for 30 min (Promega, UK) and DNA was measured by assessing remaining level of fluorescence in these wells. Nucleic acid levels were calculated from standard curves and assessed as ratios.

### Statistical analysis

Mean and standard deviations (SD) were calculated using Microsoft Excel 2007. Final biometrics, clearance rates and oxygen demand were analysed by analysis of variance (ANOVA) using SigmaPlot v 12.0 (Systat Software inc., U.K.) following Bartlett’s test for unequal variance. Data demonstrating unequal variance or non-normal distribution were analyzed with the non-parametric Kruskal-Wallis H-Test. Condition indices were arcsine transformed prior to analysis. Dry tissue and shell weight : shell height relationships were analysed on log_e_ transformed data using the general linear model using Minitab_v13_ (Minitab Inc., PA, USA). Variability and distribution of the RNA: DNA ratio data between tanks, treatments and tissues were assessed graphically. Due to the pseudo-replicated nature of the study, a hierarchical linear mixed model (LMM) was employed to account for the variability within tanks and individuals. The RNA: DNA ratio data was square root transformed prior to analysis. Treatment, tissue and the two-way interaction between these were modelled as fixed effects. Each individual animal was modelled as a random effect nested within tank, which was also modelled as a random effect. Models were built using the lme4 package [56] in R v 2.12.2 [57], p-values associated with the model point estimates were determined using the language R package [58]. Salinity, temperature, TA, DIC and derived values of pH from weekly water samples were analysed to show where there were differences between treatments or replicates. Salinity and temperature appeared to be independent observations at each time point and were analysed by 2-way ANOVA. Total alkalinity (r=0.44), DIC (r>0.95) and pH (r>0.95) were all found to be serially correlated and were analysed with repeated measures ANOVA [59]. With the exception of pH, parameters derived from CO2SYS were not analysed.

## Results

### Abiotic measurements

Measurements of pH from *in situ* electrodes and pH, as calculated by CO2SYS from TA and DIC, confirmed alteration in seawater pH following equilibration with the modified gas mixtures (Table 2). There was a relatively consistent offset between the pH logged by the electrodes and that determined from periodic water samples determined from TA and DIC by CO2SYS. The pH_(NBS)_ recorded by *in situ* probes was 0.18 units lower than pH_(seawater)_ derived by CO2SYS and agreed with the 0.18 unit difference between the calibrated electrodes and the certified seawater standard. When corrected by this offset, the continuous record of pH_(seawater)_ from the *in situ* probes was consistent with pH_(seawater)_ derived from periodic assessment of TA and DIC (Table 2). Salinity and temperature remained constant throughout the study irrespective of treatment (Salinity: F_3,91_=0.06, P>0.5; Temperature: F_3,91_=1.85, P>0.1) or replicate (Salinity: F_3,91_=0.13, P>0.5; Temperature: F_3,91_=0.56, P>0.5). Measured TA was similar between treatments (F_3,91_=0.04, P>0.5) and there were no significant differences between replicates (F_3,91_=0.13, P>0.5). However, DIC was significantly different between treatments (F_3,91_=489.33, P<0.001) and similar between replicates (F_3,91_=0.38, P>0.5). Consequently, the exposure pH as calculated by CO2SYS was significantly different between treatments (F_3,91_=842.89, P<0.001) but identical within replicate trays (F_3,91_=0.96, P>0.05). Using the Dickson and Millero parameters [49,50], the *p*CO_2_ of exposure water, as calculated by CO2SYS, differed to that of the preset equilibration gas (Table 2). This was similar across all treatments with measured *p*CO_2_ values in exposure waters between 115 µatm and 148 µatm higher than in the pre-set gas (Table 2).

**Table 2:** Water parameters measured by *in situ* probes or chemical analysis of weekly water samples, presented with parameters calculated using CO2SYS.

		Preset CO_2_ (µatm)							
		290		390		750		1140	
*in situ* probes	CO_2_ (µatm)^(a)^	287	±36	379	±34	754	±34	1180	±66
	pH_(seawater)_^(b)^	7.99	±0.02	7.95	±0.02	7.68	±0.03	7.58	±0.03
	Temperature (ºC)^(c)^	15.10	±0.20	15.11	±0.11	15.12	±0.16	15.00	±0.21
measured in water sample	Salinity (ppt.)^(d)^	34.70	±0.35	34.69	±0.35	34.73	±0.36	34.68	±0.41
	Temperature (ºC)^(d)^	15.85	±0.21	15.95	±0.14	15.91	±0.13	15.86	±0.21
	TP (µmol/L)	2.24	±0.67	2.16	±0.69	2.18	±0.65	2.14	±0.64
	TSi (µmol/L)	4.25	±0.94	4.27	±0.88	4.25	±0.98	4.25	±0.90
	TA (µmol/Kg)	2379.8	±14.8	2379.9	±14.3	2380.5	±13.2	2381.1	±13.7
	DIC (mol/kg)	2150.1	±15.3	2182.3	±23.0	2264.1	±12.2	2318.1	±13.5
Calculated by CO2SYS	pH_(seawater)_	8.026	±0.027	7.959	±0.037	7.767	±0.025	7.619	±0.032
	TCO_2_ (µmol/kg)	16.02	±1.14	19.19	±2.05	31.50	±1.92	45.77	±3.66
	*p*CO_2_ (µatm)	414.7	±31.7	528.0	±55.8	866.0	±52.8	1255.9	±101.8
	Calcite saturation	4.01	±0.21	3.54	±0.25	2.40	±0.13	1.76	±0.12
	Aragonite saturation	2.58	±0.13	2.28	±0.16	1.54	±0.08	1.13	±0.08
	HCO_3_^-^ (mmol/L)	1965.9	±19.9	2014.8	±29.9	2131.9	±13.3	2198.6	±13.4
	CO_3_^2-^ (mmol/L)	168.15	±8.70	148.25	±10.46	100.68	±5.33	73.73	±4.98

Values presented as mean values with standard deviation (±SD), Total alkalinity (TA), total phosphate (TP), total silicate (TSi), dissolved inorganic carbon (DIC), total CO_2_ (TCO_2_), partial pressure of CO_2_ (*p*CO_2_); bicarbonate (HCO_3_^-^), carbonate (CO_3_^2-^)

a equilibration gas measured using calibrated Li-COR gas analyser averaged over a minimum of 30min.

b pH recorded on NBS scale in exposure tanks at 10min intervals (n >12000) and converted to seawater scale by applying a - 0.181unit offset.

c temperature recorded using electrodes in selected exposure tanks at 10min intervals (n >12000).

d salinity or temperature of water samples at point of sampling for TA and DIC (n=23).

### Biometrics

**Figure 1:**
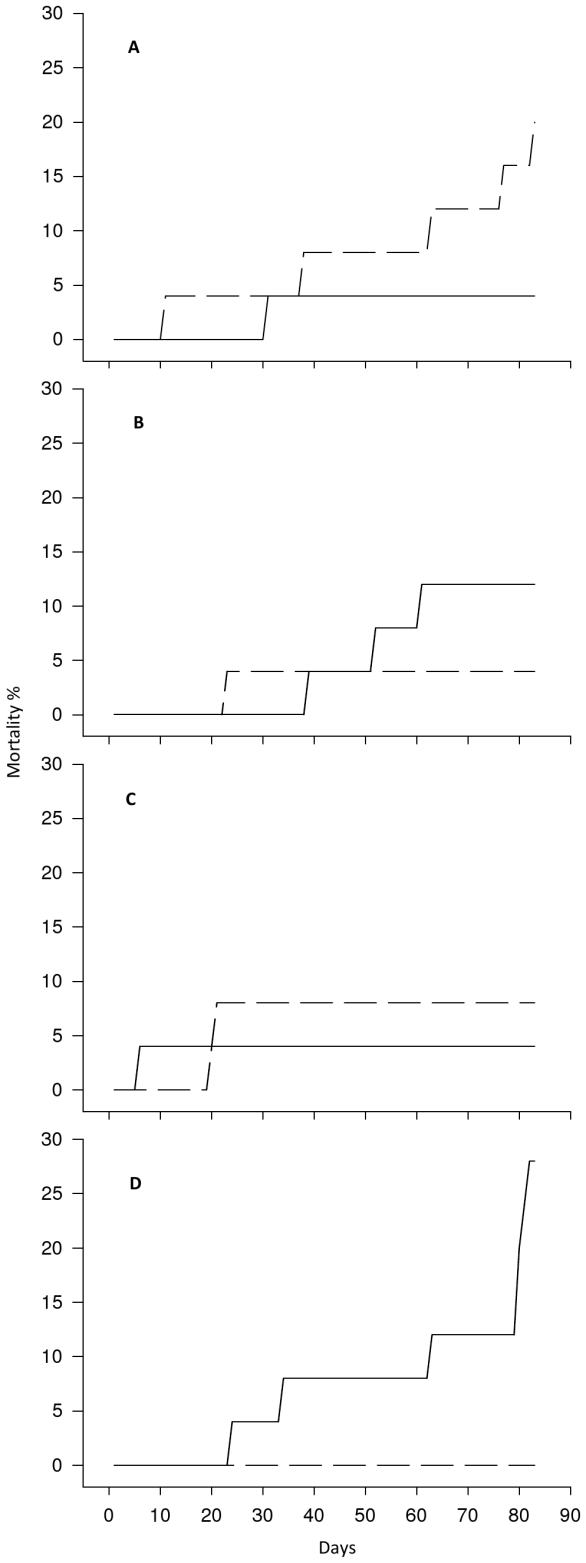
Effect of *p*CO_2_ on cumulative mortality of *P. maximus* over 82 days. Initial stocking 25 scallops per tray under *p*CO_2_ treatment 290(A), 390 (B) 750(C) 1140 (D) µatm. Solid lines are mortality in replicate A, hashed lines replicate B.

**Figure 2:**
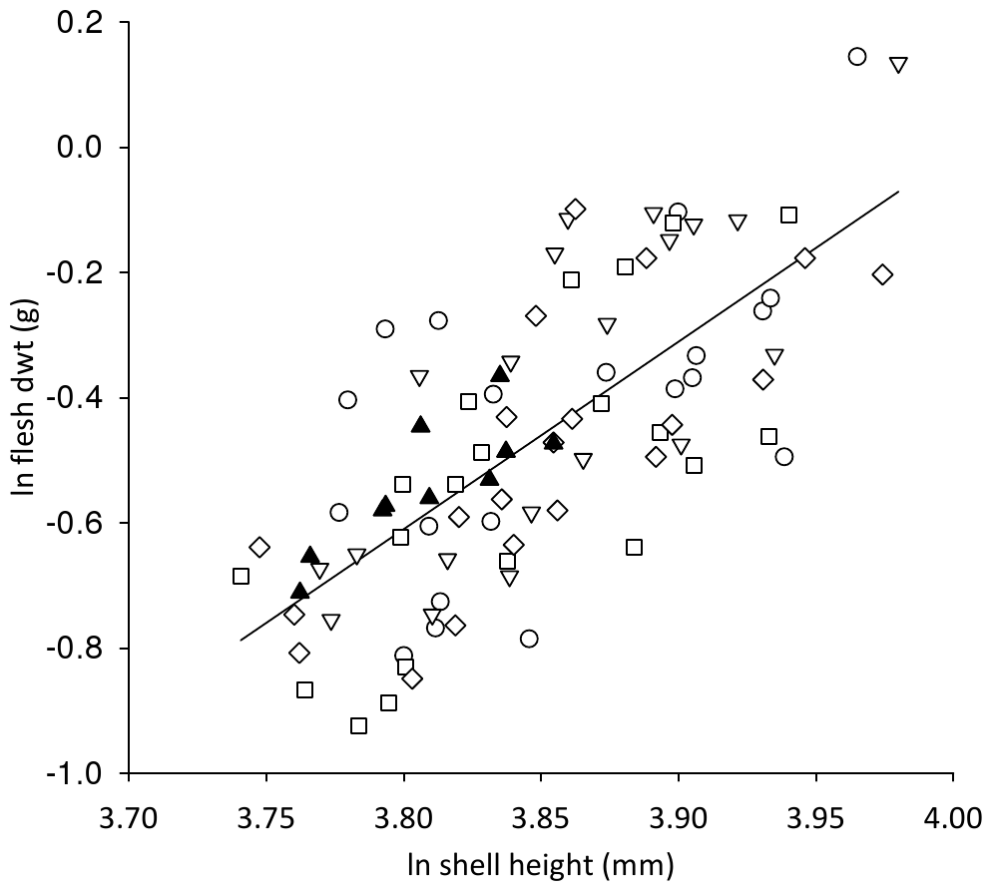
Effect of *p*CO_2_ on the flesh weight : shell weight relationship of juvenile *P. maximus.* Logarithmic plot of scallop flesh dry weight (d wt) against shell height illustrating the similar relationship (Ancova: F_1,80_=2.37, P>0.05) between untreated scallops on day 0 (filled triangle) compared with individuals exposed to *p*CO_2_ 290(open circle), 390 (open triangle), 750 (open square) 1140 µatm (open diamond) for 82 days; regression line fitted against all data.

**Figure 3:**
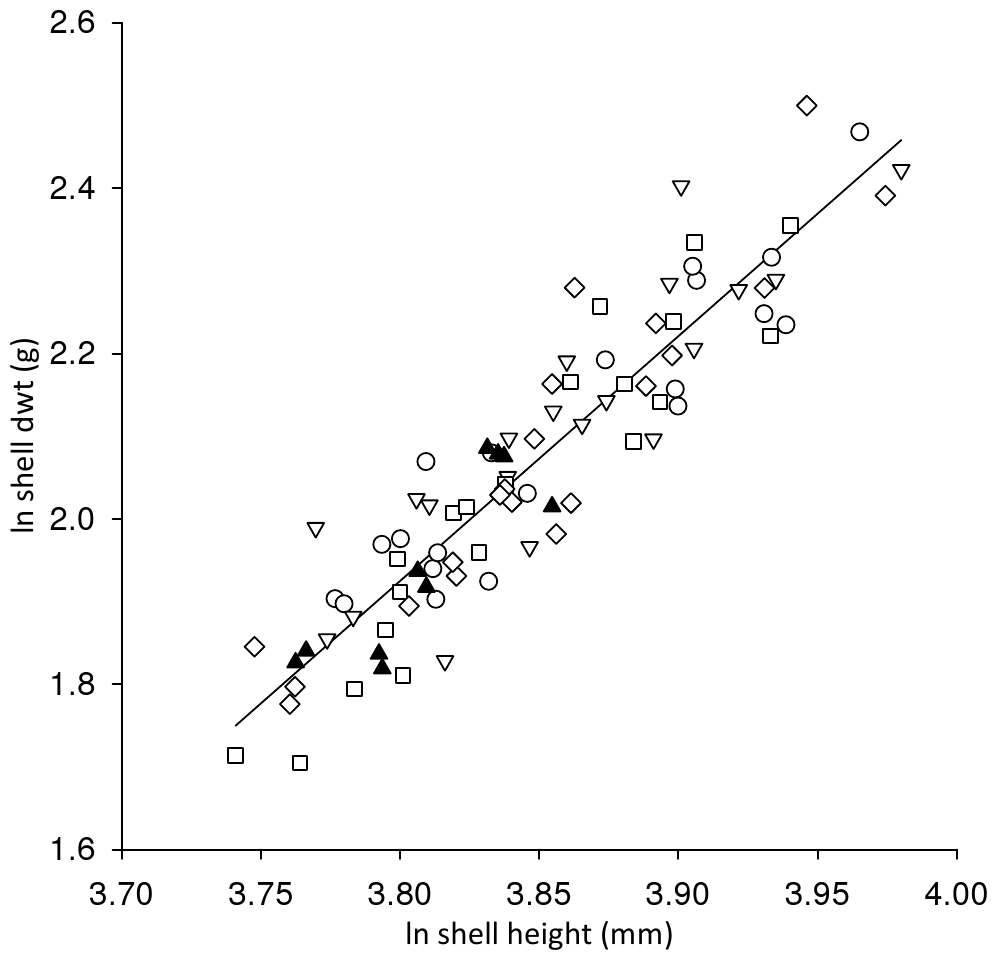
Effect of *p*CO_2_ on the shell height: shell weight relationship of juvenile *P. maximus.* Logarithmic plot of shell dry weight (d wt) against shell height (mm) illustrating the similar relationship (Ancova: F_1,80_=0.48, P>0.05) between untreated scallops on day 0 (filled triangle) compared with individuals exposed to *p*CO_2_ 290(open circle), 390 (open triangle), 750 (open square) 1140 µatm (open diamond) for 82 days; regression line fitted against all data.

Scallop mortality was low up to day 78 (4-12%), after which there was a slight increase in mortality in single replicates of the 290 and 390 µatm treatments (Figure 1). The condition index of the scallops sampled at the end of the study (Table 3) did not significantly differ between replicates (F_4,81_= 2.12, P>0.05) nor from scallops sampled prior to exposure (F_4,85_=1.00, P>0.05). There was no significant difference (H = 4.567, d.f. 3, P>0.05) in the growth of the scallop shells between treatments, as measured by shell height (Table 3). There was no significant difference (H=6.385, d.f. = 8, P >0.05) in the clearance rates of scallops prior to, and following, treatment (Table 3). With an increase in shell height there was a significant increase in both shell dry weight (Ancova: F_1,80_=265.19, P<0.001; β=2.65, t=9.93, P<0.001) and flesh dry weight (Ancova: F_1,80_=45.22, P<0.001; β=2.93, t=21.66, P<0.001). The relationship between the shell height and the tissue dry weight (Figure 2) was not significantly different between treatments nor significantly different to scallops at the start of the exposure (Ancova: F_4,80_=0.48, P>0.05). The shell height: shell dry weight relationship (Figure 3) remained constant between treatments and was not significantly different to scallops at the start of the exposure (Ancova: F_1,80_=1.08, P>0.05). There was no significant difference (F_8,39_=1.150, P>0.05) in the measured oxygen consumption of pre-treated scallops and those exposed in any of the replicated treatments (Table 3).

**Table 3:** Condition index, shell height difference, clearance rate and oxygen demand of scallops prior to (Day 0) and following exposure to pH modified seawater.

	Condition index		shell height difference (mm)		Clearance rate (L/h/g dw)		Oxygen Demand (µmolO_2_/h/g dw)	
	mean	SEM	mean	SEM	mean	SEM	mean	SE
Day 0	0.084	0.002	-	-	5.954	0.675	16.330	1.343
290 (A)	0.074	0.003	0.163	0.127	5.551	0.631	17.226	1.779
290 (B)	0.088	0.006	0.325	0.061	6.076	0.925	17.921	0.834
390 (A)	0.082	0.004	0.106	0.122	6.373	0.609	18.435	1.363
390 (B)	0.085	0.005	0.276	0.137	6.873	0.969	17.737	1.018
750 (A)	0.074	0.003	0.131	0.059	5.324	0.745	17.003	1.586
750 (B)	0.081	0.004	0.179	0.052	6.328	0.508	16.244	1.020
1140 (A)	0.079	0.003	0.120	0.073	7.211	0.637	20.340	1.056
1140 (B)	0.076	0.003	-0.027	0.079	6.038	0.688	19.826	1.555

Values presented as mean values with standard error (SEM). Condition index n=10; shell height difference n=20 except 1140 (A) where n=18; Clearance rate n=5 except Day 0 where n=10; Oxygen demand n=5, except Day 0 where n=8.

### Biochemical indicators of growth

**Figure 4:**
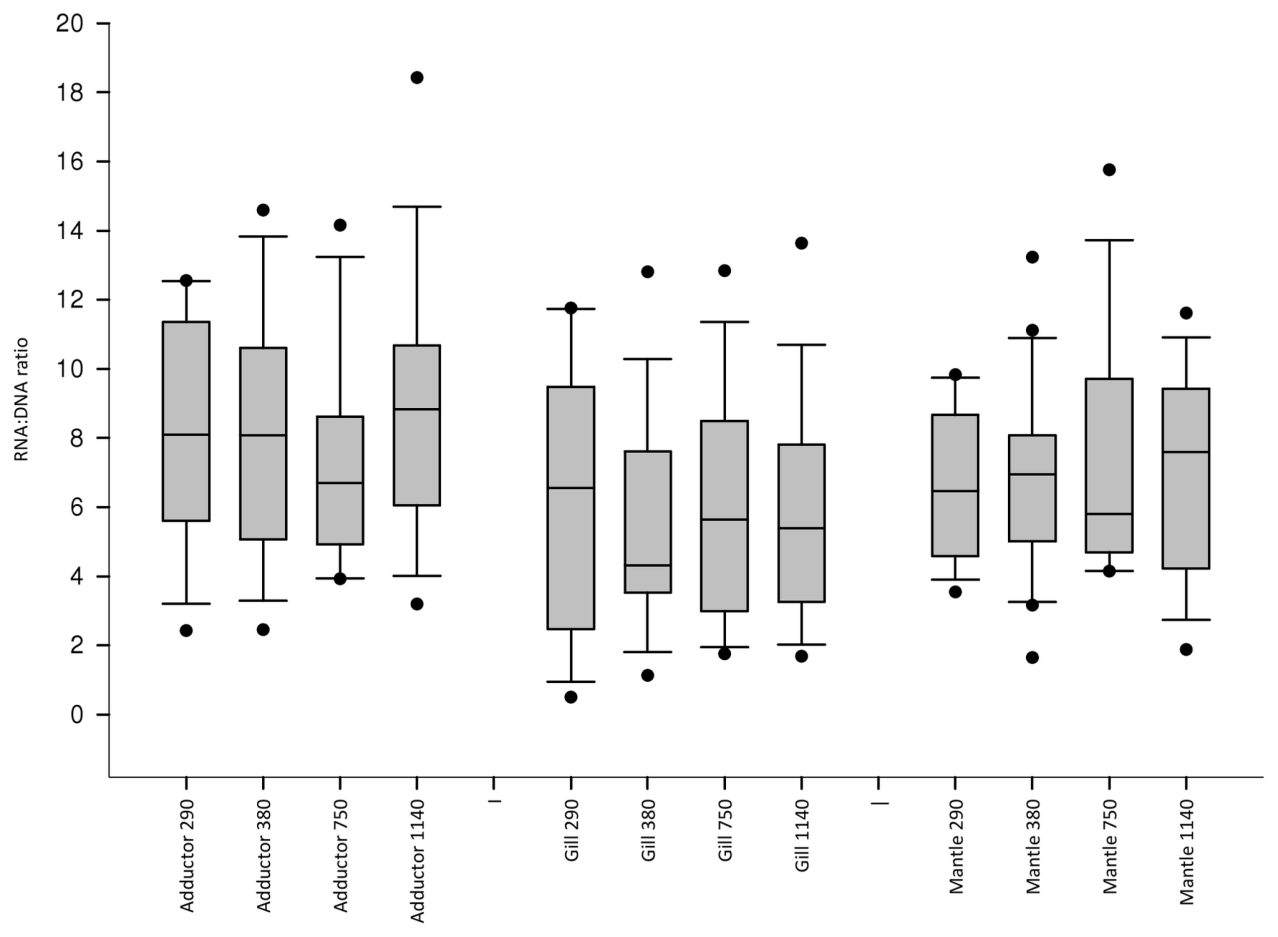
Effect of *p*CO_2_ on the RNA: DNA ratio of juvenile *P. maximus.* RNA: DNA ratios are presented in adductor muscle, gill and mantle tissue denoted on the X axis in preset *p*CO_2_ (µatm) (left to right n=6,17,18,17,16,16,17,19,16,17,20,17).

**Figure 5:**
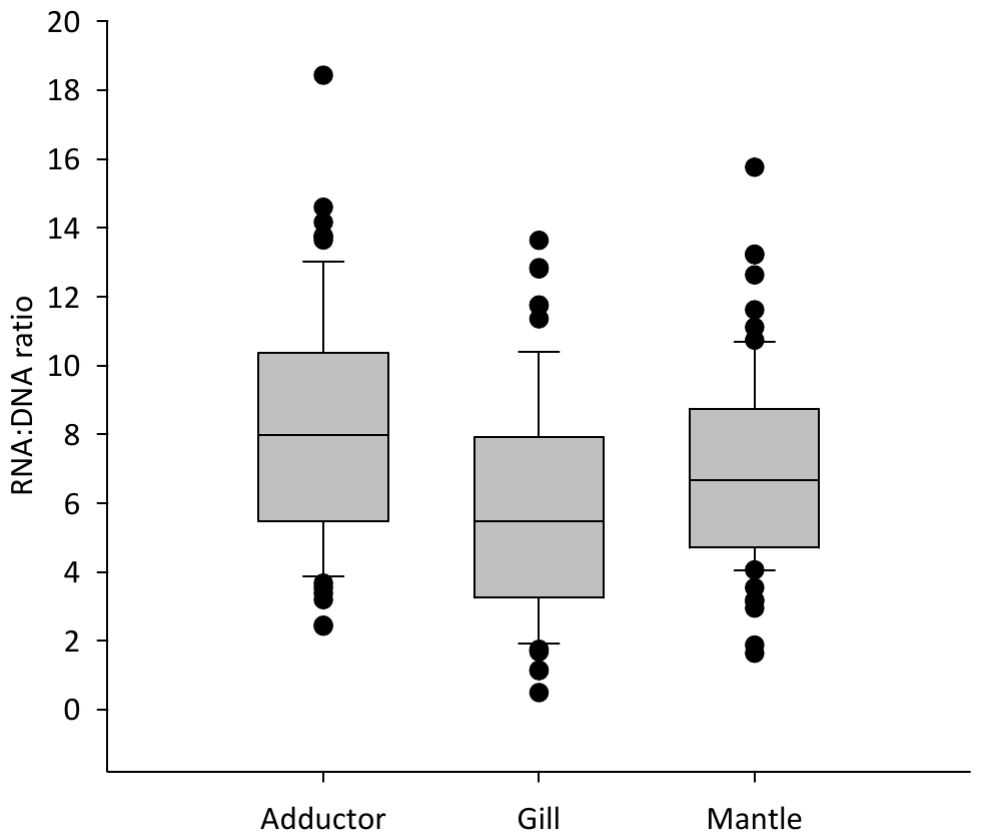
RNA: DNA ratio of juvenile *P. maximus* by tissue type. Adductor RNA: DNA ratio is significantly higher than that of gill (p<0.001) or mantle (p = 0.028). n= 70 for mantle, n= 68 for both adductor and gill.

The mean RNA: DNA ratio by tissue type and treatment is presented in Figure 4. The LMM demonstrated that none of the treatment levels studied had a significant influence on the RNA: DNA values observed (F=0.074, d.f. = 3, sum of squares=0.092, mean squares=0.031, p=0.974) and that there was no significant interaction effect between treatment and tissue type (F=0.065, d.f. = 6, sum of squares=0.162, mean squares=0.027, p=0.999). These terms were removed from subsequent models and tissue type alone was retained as it explained a significant amount of the observed variability (F=10.276, d.f. = 2, sum of squares=8.554, mean squares=4.277, p<0.001). The RNA: DNA ratios by tissue alone are presented in Figure 5. Both gill (β=-0.501, S.E.=0.109, t=-4.600, p<0.001) and mantle tissues (β=-0.245, S.E.=0.108, t=-2.270, p=0.024) were found to have significantly lower RNA: DNA ratios than adductor muscle. Gill tissue was also found to have a significantly lower RNA: DNA ratio than mantle (β=-0.256, S.E.=0.108, t=-2.237, p=0.019).

## Discussion

This study maintained king scallops, *Pecten maximus*, under pH conditions ranging from 8.03–7.62 (Δ -0.07 to -0.4) to assess physiological impacts at the whole organism level. Critical to conducting and interpreting the experimental data is close control and monitoring of carbonate parameters in the experimental system [44]. Analysis of total alkalinity (TA) and dissolved inorganic carbon (DIC) data indicated pH conditions were within the intended range, but *p*CO_2_ was typically 115 µatm higher than in the equilibration gas. Since TA and DIC measurements were within the 0.1% recommended by Riebesell et al. [44] and the calibration gases are certified to ±1%, CO_2_ contributed from biological activity in the exposure trays and possible biofilms in the equilibration columns is the most likely explanation for the discrepancy. Overall the exposure regime in this study remained consistent with the revised representative concentration pathways for CO_2_ emissions up to 2100 [60].

Shell heights and condition indices of the scallops at the end of the study were not significantly different to those at the start, irrespective of treatment. Although the feeding regime in this study was high in comparison to similar studies [25], shell growth rates, as measured by shell height, were negligible. When feeding on natural seston, the somatic and shell growth of both juvenile *Mercenaria* *mercenaria* and *Argopectenirradians* are unaffected by pH reductions of ≤0.45 units (1700 ppm *p*CO_2_) but shell growth of juvenile *Crassostrea* *virginica* is reduced; though somatic growth unaffected [61]. During this study, scallops were fed an artificial diet of mixed algae at cell concentrations around 100 cells µl^-1^, approximately 4.45 mg L^-1^. These cell numbers are up to 5 times higher than those in similar studies with adult *Mytilusgalloprovincialis* fed live algae (3.84 mg L^-1^) [35]. Laing [42] demonstrated that in homologous cultures of algae ( *Pavlova* *lutheri* or *Chaetoceroscalcitrans* ) the filtration rate of *P* *. maximus* was largely unaffected up to 200 cells µl^-1^. However, in heterogenous cultures (as during the exposure phase), filtration of *P* *. maximus* has been found to vary with algal cell size (2.7-8.1 µm) and filtration rates are inversely proportional to cell concentrations between 8-100 cells µl^-1^ [42]. Using Shellfish Diet 1800 at ~40 cells µl^-1^, Bechmann et al. [62] reported significant growth in *Mytilus* *edulis* larvae over the first 64 days post fertilisation and when exposed to seawater reduced in pH by 0.43 units (~1400 µatm) the overall larval growth was restricted. The lack of any significant change in the condition indices of scallops in this study, compared with animals at the start, indicates that the animals were feeding at a rate sufficient to maintain somatic tissue but not sufficient to promote shell or somatic growth.

A similar study with *P* *. maximus* using another artificial diet, observed no significant changes in the condition index or growth of *P* *. maximus* at 10°C due to acclimation to similar OA conditions (1100 µatm) but at lower temperatures (4°C) the authors observed a significant increase in mortality [25]. During *P* *. maximus* larval development, exposure to *p*CO_2_ levels elevated by 1150 µatm above ambient, reduces shell growth by 5% and survival by 30% [63]. In the present study, although an increase in juvenile mortality occurred in single replicates during the last 4 days of the study, the mortality observed throughout the study (<12%) were generally similar to the 5-10% reported in studies with other juvenile bivalves [35].

The clearance rate (CR) and assimilation efficiency, combined with the nutritional content of the diet, provides a direct measure of energetic intake of most bivalves [34]. Consequently, assessment of CR on its own provides a good indicator of physiological status of bivalves. Short term studies with various species of bivalves have reported differences in the sensitivity of the CR to altered pH. Liu and He [26] reported reductions in the clearance rates of both the clam, *Chlamys nobilis*, and the mussel *Perna* *viridis* when held for 5 days under pH conditions similar to this study (Δ-0.4 units). Conversely, the clearance rate of the pearl oyster held under identical conditions was reported to increase, though variance in the data appeared anomalous [26]. Medium term studies (>60 d) under similar conditions (Δ ≤-0.4 units) have reported reductions in the CR of the clam *Ruditapes* *decussatus* [9] and the mussel, *Mytilus* *chilensis* [64]. In contrast, clearance rates of *M* *. galloprovincialis* remained unaffected by pH reductions of 0.6 units (~3800 µatm *p*CO_2_) [35]. Bechmann et al. [62] observed no effect on the feeding of *M* *. edulis* larvae using *I* *. galbana* *fed* at the same concentration as an artificial diet (Shellfish 1800) fed during the exposure period. Adopting a similar approach, this study found no significant effect of reduced pH (Δ -0.4) on the ability of juvenile *P* *. maximus* to filter an homogenous algal suspension of *I* *. galbana* between 20–100 cell µl^-1^. Juvenile *P* *. maximus* has demonstrated the ability to selectively filter algal cells based on size [42] and it remains to be investigated whether this discrimination is affected by pH. Such an effect could have significant implications for the nutritional intake of wild scallops feeding on natural seston.

Numerous studies investigating the impact of ocean acidification on marine bivalves have focussed on shell deposition (calcification) and growth [19,22,65]. Using the alkalinity anomaly method, Waldbusser et al. [20] reported minimal effects on calcification rates of two species of *Mercenaria* spp. with all but the smallest individuals (<0.4 mm) able to maintain calcification rates sufficient to prevent net shell dissolution at *p*CO_2_ levels similar to those in this study. Findley et al. [66] reported increased calcification rates of some brittlestar and limpet species under reduced pH while declining calcification rates in *Chlamys farreri* were found to be directly correlated with declining pH and rising *p*CO_2_ [67]. This study did not measure the specific rate of calcification in scallops. However, the various treatments had no significant effect on the shell height: shell weight ratio at the end of the study and this relationship remained the same for individuals prior to exposure. Consequently, no detectable net calcification could be identified.

Mechanisms like oxygen supply and respiration can respond strongly to alterations in surrounding CO_2_ conditions [36,68,69]. The respiration rate of the clam, *C* *. nobilis* , has been shown to be unaffected by pH reductions of ≤0.4 units during short term exposure (<5 d) but at Δ-0.7 units respiration is significantly reduced [26]. Increased respiration in the Antarctic clam, *Laternula* *elliptica* , has been observed during medium term exposures (80-120 d) under conditions where the pH has been reduced by as little as 0.22 units [70]. Conversely, decreased respiration has been observed in the commercially important calm, *R* *. decussatus* , at ≤-0.4 units [9]. Species differences in the respiratory response of mussels and oyster have also been reported. During very short studies (2 h) the respiration rates of both *M* *. edulis* and *Crassostrea* *gigas* juveniles/adults were unaffected by *p*CO_2_ levels up to 2000 µatm (Δ -0.7 units) [19]. Longer term studies with oysters have observed no effect on the respiration of *C* *. gigas* following exposure to pH reductions of 0.4 units [71] but aerobic metabolism in *C* *. virginica* was reduced at ≥-0.7 units[72]. A more direct comparison was observed with *M* *. galloprovincialis* where reductions of ≤0.6 units had no significant effect on respiration [9] but reductions of ≥0.7 units did [7]. In contrast to M. *galloprovincialis*, an increase in the respiration rate of *M* *. edulis* was observed at only -0.3 units [39].

With differing feeding regimes, acclimation duration, methodology and test species it is difficult to identify the reason for differing metabolic responses between studies. Studies on calcification within species have identified differing responses based on population and individual size [20] but differing responses have been largely identified between species [16,26]. In this study, the resting metabolic rate (RMR) of scallops exposed to *p*CO_2_ concentrations of 500-1200 µatm, as measured by O_2_ consumption, did not differ significantly to unexposed individuals at the start of the study. Schalkhauser et al. [25] also observed no significant difference in the RMR of *P* *. maximus* following exposure to similar *p*CO_2_ conditions at 10 °C but did observe differences in the maximum metabolic rate (MMR) following exercise. Although the MMR of *P* *. maximus* was not assessed in this study, the results from the two studies with *P* *. maximus* conducted at different temperatures, under differing feeding regimes and from different populations, suggest that changes in seawater pCO_2_ ≤1140 µatm will have no adverse impact on the RMR of *P* *. maximus* .

In addition to metabolic depression, changes in ammonium (NH_4_^+^) excretion rates have been observed in *M* *. edulis* exposed to *p*CO_2_, >2000 µatm [18]. When pH was reduced by 0.4 units, Liu and He [26] observed reductions in the ammonia excretion rates of *Pinctada* *fucata* and at -0.7 units similar reductions were observed in *C* *. nobilis* and *P* *. viridis* . At very high *p*CO_2_ levels (>4000 µatm), the inverse correlation between NH_4_^+^ excretion and O:N ratios, indicative of enhanced protein metabolism, suggests that inhibition of shell growth arose from increased cellular energy demand and nitrogen loss [18]; not through metabolic depression as suggested by Pörtner et al. [73]. This increased ammonium excretion arising from elevated protein catabolism could be a means of internally producing bicarbonate (HCO_3_) in an attempt to regulate pH [24,74]. However, the limited ability of bivalves to regulate internal acid–base balance [18,25,70] suggests that alterations in metabolic partitioning may be the main effect of OA. It was not possible to measure ammonium excretion rates of scallops exposed in this study to pH changes of -0.4 units. However, Fernández-Reiriz et al. [9] observed an increase in the excretion rate of the clam *R* *. decussatus* when pH was decreased ≥0.4 units and an increase in the excretion of *M* *. galloprovincialis* at a decrease of ≥0.6 units [35]. Similar species differences were observed in short term studies by Liu and He [26] using pH adjusted seawater ≥ -0.4units. These data suggest that it should be considered that alterations in the excretion rates of scallops in this study may not have been observable under the *p*CO_2_ conditions tested (Δ ≤-0.4 units).

Nucleic acid-derived indices are widely used in marine ecology [75], with RNA: DNA ratios used in fish and invertebrates as an indirect measure of growth [76–78], nutritional condition [79] and health status following exposure to environmental contaminants [80–82]. The few studies measuring RNA: DNA ratio in bivalves have primarily used it as a means of assessing nutritional condition and changes in RNA: DNA ratio have been reported arising from starvation or poor nutritional status [75,83]. Lodeiros et al. [84] reported that the RNA: DNA ratio is correlated with muscle growth in the scallop *Euvolaziczac* but inversely correlated with stress. The RNA: DNA status of the animals on day 0 was not available for this study, but the lack of treatment effect on the RNA: DNA ratio in any tissue type following exposure is supported by the lack of similar effects on the condition index [85]. At the end of this study RNA: DNA ratios from the different *P* *. maximus* tissues were significantly different from each other, as would be expected [83], but were unaffected by the various acidified water treatments. This suggests that the treatment had no significant effect on the growth potential of juvenile *P* *. maximus* and that the animals were feeding at a level sufficient to maintain somatic tissue.

A limitation of this and other studies in the literature involving OA and bivalves is that no histopathology examination of animals was undertaken prior to the study. The disease status of an organism is likely to influence their ability to respond to environmental stressors [86] and the results of Bibby et al. [12] suggest that the immune function of bivalves may be impacted by OA; possibly through increased energetic cost to the animal. In this study the lack of discernible effects between the treatments suggest that any sub-clinical infection did not alter the organism’s ability to tolerate exposure to increased acidity, though it is recommended that future investigation consider the role that disease and parasites play on the tolerance of organisms to OA.

Global average surface seawater pH is currently around pH 8.2 and naturally fluctuates by more than ±0.3 pH units depending on region, season and phytoplankton activity [87]. The OA exposure scenarios in this study reflect those predicted for oceanic waters [4,5] and are comparable to the worst case scenario (~1000 ppm CO_2_) modelled by Blackford and Gilbert [88] for North Sea waters where a decrease of ~0.4 units is predicted by 2100. In contrast, relatively large variations in seawater pH have been reported in coastal areas [89], with Yu et al. [90] observing variances of more than 0.3 units within 24 h. In some coastal environments, natural surface water *p*CO_2_ levels of 375-2300 µtm have been observed [18] and under hypoxic conditions levels as high as 3200 µtm are not unexpected in coastal environments [91].

The initial predictions of detrimental effects on calcification of juvenile/adult *M* *. edulis* under future OA scenarios [19], contrast with recent studies [18,92] indicating that this species, at least, is capable of inhabiting environments where natural *p*CO_2_ levels are 1000-4000µatm. In many published studies, depressed metabolic rates and inhibition of feeding activity has been reported in bivalves at pH levels ≥-0.7 units [36] and such impacts occur at the limits predicted by climate models for changes in the open ocean. This may not be relevant for coastal environments. Further studies using exposures as high as 4000 µatm *p*CO_2_, and considering interactions of *p*CO_2_ with hypoxia [91,93] may provide a more realistic insight into the impact of OA on the physiological responses of bivalves in coastal environments.

A recent study by Thomsen et al. [92] indicated that *p*CO_2_ has only a minor effect on the growth and calcification of juvenile *M* *. edulis* and that food availability is a primary factor driving biomass and biogenic CaCO_3_ production. Although the intention was not to provide food limiting conditions in this study some factor/s prevented somatic and shell growth and presented sub-optimal conditions for the scallops during the exposure period. Effects of OA on tolerant species are most likely to be observed at points of physiological thresholds or under conditions not favouring optimal growth [68]. Furthermore, organisms may have less capacity to respond to environmental stressors when they are already under food limiting conditions [92,94]. Results from this study indicate that exposure of juvenile scallops for 3 months to *p*CO_2_ levels up to 1200 µatm (Δ-0.4 units), under potentially sub-optimal conditions, has little effect on basal aerobic metabolic demand or the clearance rate of juvenile *P* *. maximus* ; two key parameters in energy partition models.

The lack of effect on basal metabolism and clearance rates in this study is in contrast to adverse effects reported in marine animals encountering co-stressors. Schalkhauser et al. [25] reported a restriction in the thermal tolerance of *P* *. maximus* and noted a reduction in the force exerted by the adductor mussel during the escape response at *p*CO_2_ levels of ~1100 µatm. The effect on escape response suggests that behavioural endpoints could be an important consideration in assessing detrimental effects of OA in invertebrates as well as in fish [95]. Furthermore, Roberts et al. [43] observed an increase in the toxicity of marine sediments when the pH of overlying seawater was reduced by 0.4 units. These findings support the premise that the impacts of OA will most likely be apparent at physiological extremes. Further work should focus on the impacts of realistic exposure based on conditions in coastal habitats and food availability [92,94] and consider the interaction of acidification with co-stressors like hypoxia [91,94] temperature [25,71] or contaminants [43].

The recent study by Andersen et al. [63] indicates that the growth, development and survival of the initial larval stages of *P* *. maximus* are susceptible to the effects of OA at *p*CO_2_ levels of ≥1600 µatm. In contrast, the lack of observable effects in this study, even under sub-optimal conditions, suggests that when food availability is not a limiting factor, juvenile *P* *. maximus* display some tolerance to pH reductions of ≤0.4 units. However, further studies under known food limiting conditions or at higher *p*CO_2_ levels may show *P* *. maximus* to be just as susceptible to the effects of OA as has been reported for other bivalve species.
